# Development and evaluation of a new measure of children’s play: the Children’s Play Scale (CPS)

**DOI:** 10.1186/s12889-021-10812-x

**Published:** 2021-05-07

**Authors:** Helen F. Dodd, Rachel J. Nesbit, Laura R. Maratchi

**Affiliations:** School of Psychology and Clinical Language Science, University of Reading, Harry Pitt Building, Whiteknights Road, Earley Gate, Reading, RG6 6ES UK

**Keywords:** Play, Children, Child health, Risky play, Adventurous play, Questionnaire, Measure, Survey

## Abstract

**Background:**

There is increasing recognition of the importance of children’s play from a public health perspective, given the links between play and children’s physical and mental health. The present research aimed to develop and evaluate a new parent-report questionnaire that measures the time children spend playing across a range of places and includes a supplement to evaluate how adventurously children play.

**Methods:**

The questionnaire was developed with input from a diverse group of parents and experts in children’s play. It was designed to yield a range of metrics including time spent playing per year, time spent playing outside, time spent playing in nature and level of adventurous play. The reliability of the questionnaire was then evaluated with 245 parents (149 mothers, 96 fathers) of 154 children aged 5–11 years. All participants completed the measure at time 1. At time 2, an average of 20 days later, 184 parents (111 mothers and 73 fathers) of 99 children completed the measure again.

**Results:**

Cross-informant agreement, evaluated using Concordance Correlation Coefficients (CCCs), ranged from 0.36 to 0.51. These fall in the poor to moderate range and are largely comparable to cross-informant agreement on other measures. Test-retest reliability for mothers was good (range 0.67–0.76) for time spent playing metrics. For fathers, test-retest reliability was lower (range 0.39–0.63). For both parents the average level of adventurous play variable had relatively poor test retest reliability (mothers = 0.49, fathers = 0.42). This variable also showed a significant increase from time 1 to time 2. This instability over time may be due to the timing of the research in relation to the Covid-19 lockdown and associated shifts in risk perception.

**Conclusions:**

The measure will be of value in future research focusing on the public health benefits and correlates of children’s play as well as researchers interested in children’s outdoor play and play in nature specifically. The development of the measure in collaboration with parents and experts in children’s play is a significant strength. It will be of value for future research to further validate the measure against play diaries or activity monitors.

**Supplementary Information:**

The online version contains supplementary material available at 10.1186/s12889-021-10812-x.

## Background

There is increasing interest in children’s play from a public health perspective [1, 2]. Time outdoors and children’s physical play, which includes jumping, climbing, swinging, balancing and running, encourage physical activity which supports obesity prevention and promotes good physical and mental health more broadly [3–6]. There are concerns that children’s outdoor play has declined in recent decades [7, 8] and that this may have significant implications for children’s health and wellbeing [9]. In particular, declines in adventurous play, where children are allowed to take risks and challenge themselves during play, may have implications for mental health [6, 10]. Despite this, research on the public health sequalae of children’s play (or lack thereof) is rare, particularly in relation to mental health [3]. To conduct research of this nature we require instruments that can capture children’s play on a large scale. Quantifying children’s play experiences on a public health scale is challenging and few instruments currently exist to support this. In this article we therefore present the development and evaluation of a new parent-report measure that first estimates children’s time spent playing in a range of places and includes a supplement that estimates their level of adventurous, or risky, play.

Following concerns about children’s levels of physical activity, Veitch and colleagues developed a parent-report measure of children’s outdoor play [11]. In this measure, parents are asked to report the number of days their child spent playing in each of eight locations in a typical week. Parents are instructed to include a day in the total if their child had spent at least 10 min in the relevant location. Test-retest reliability was examined using Intra-Class Correlation (ICC) and ranged from poor (ICC = 0.18) to good (ICC = 0.82), with an average ICC of 0.58. Responses were also compared to an activity logbook that parents kept over the course of a week prior to completing the measure. Some evidence of moderate consistency between the measure and the logbook was found. Subsequent research has examined associations between children’s outdoor play, using this measure, and their physical activity and has failed to find the expected association [12]. One reason for this might be that the measure does not capture the total amount of time children spend playing outdoors, only the frequency with which they play outdoors.

The amount of time children spend playing outdoors is the focus of The Outdoor Playtime Checklist [13]. When completing this measure, parents respond to two items asking how much time their child spends playing outside home during three different periods of the day (wake-up – noon, noon – 6 pm, 6 pm- bedtime), using a 5 point-interval scale, ranging from 0 min to over 60 min. The two items refer broadly to play outside at home/outside at others’ homes and play outside away from home (park, playground, recreation area). Unlike the measure by Veitch and colleagues [10] above, time spent playing outdoors on this measure was found to be modestly associated with physical activity measured using an accelerometer (*r* = 0.33, *p* < .001), implying that time spent playing is more closely related to physical activity than frequency of play.

Both existing measures described have strengths; the measure developed by Veitch and colleagues [11] captures information on where children play and the Outdoor Playtime Checklist [13] captures time spent playing. Neither measure though captures the time that children spend playing in different places. A number of measures have also been designed for use within individual studies to capture the time children spend outdoors. For example the Avon Longitudinal Study of Parents and Children asked parents to report how much time children typically spent outdoors on weekdays and weekend days during winter and summer [5]. Similarly, time spent outside during warmer months and cooler months after school and at weekends was evaluated via parent report in a large study focused on children’s obesity [14]. Building on the strengths of these previous measures as well as the measures of children’s play, the primary aim of the present study was to develop a parent-report measure that estimates both how often their child plays in certain places as well as how long their child plays for in each place, with a view to better capturing overall time spent playing in different places.

A secondary aim was to develop a supplement that captures how adventurously children play in each place. Adventurous play, also known as risky play, is defined as ‘exciting, thrilling play where the child experiences a level of fear and is able to take age appropriate risks’ [6, 15]. It has been proposed that adventurous play may provide children with important learning opportunities that help prevent fears and anxiety. For example, Sandseter and Kennair [16] hypothesise that children’s risky play provides an opportunity for children to be exposed to stimuli that they are developmentally programmed to fear. For example, a fear of heights is developmentally normal and may be overcome by playing at heights, perhaps in a tree or on monkey bars. Dodd and Lester [6] extend these ideas to argue that adventurous play offers children an opportunity to learn about uncertainty, coping, physiological arousal and fear. In turn they argue that this learning might help decrease children’s risk for elevated anxiety in the long term. These ideas are also compatible with the theoretical arguments made by Gray [10] who argues that through risky, adventurous play, children learn to solve their own problems, control impulses, and regulate their emotions.

Quantitative research evaluating these hypotheses around the benefits of adventurous play is rare. A review of the health benefits of risky play was conducted in 2015 identified no studies that evaluated mental health outcomes [3]. One of the obstacles to research on adventurous play is that no measures exist that allow this type of play to be quantified. A number of related measures exist, including measures of parent attitudes and behaviours around risk taking in children’s play [17, 18] as well as measures of outdoor play more generally as described above [11] but these measures do not allow children’s engagement in adventurous play to be measured.

To our knowledge, only one published study has attempted to quantify children’s participation in adventurous play in any detail. In the New Zealand State of Play Survey, Jelleyman and colleagues [19] asked parents to report the frequency with which their children engaged in specific examples of play on a 5-point Likert Scale from “Never” to “Always”. The examples were drawn from categories of risky play as defined by Sandseter [15] along with two additions (loose parts and messy play). Whilst this measure provides useful insights into adventurous play, it has a number of limitations. First, the level of adventure/risk taking is not evaluated, parents simply report how often the child engaged in each type of risky play. This is important because it may miss differences between children in how they play. For example, two children could both be playing with loose parts but one might play imaginatively with a neat pile of sticks whilst another might build a precarious construction to climb on; both are playing with loose parts but the level of risk differs significantly. Second, the frequency categories were not objectively anchored; one parent’s interpretation of “sometimes” may be very different to another’s. Third, the measure tells us nothing about where this type of play takes place. This is important for the development of programmes that aim to increase children’s adventurous play.

The two aims of this research were:

1. to develop and evaluate a parent-report questionnaire that could be used to quantify school-aged children’s time spent playing in a range of places.
2. to develop and evaluate an extension of that measure which asks parents to estimate their child’s engagement in adventurous play in each place.

The measure was developed with input and feedback from parents and experts on children’s play. It yields a range of metrics that could be used in future research, with or without the adventurous play supplement. The test-retest reliability and cross-informant reliability of these metrics are evaluated.

## Methods

### Participants

Participants were recruited via social media. The advert asked for Mums and Dads with children aged 5–11 years to complete an online survey about what their children do in their spare time. Participants were 245 parents (149 mothers and 96 fathers) of 154 children (78 boys, 76 girls) aged 5 to 11 years (*M* = 99 months, *SD* = 25 months) at baseline. Due to an oversight at time 1, child ethnicity was only collected at time 2 where the children were described as being White British (87.9%), Mixed race (6.0%), White European (2.6%), Asian or Asian British Indian origin (0.9%), Asian or Asian British, other Asian origin (0.9%) and Mixed white background (0.9%). Ethnicity was not provided for one child. For 91 children, both parents participated, for the remaining 63 children, only one parent participated. Mean parent age was 38 years (*SD* = 6 years). The majority of parents were married (*n* = 196), approximately half had university level qualifications (*n* = 123, 2 declined to answer) and the majority worked full-time (*n* = 143) or part-time (*n* = 76). All but ten parents stated that they were the child’s primary caregiver (*n* = 134) or shared primary caregiving (*n* = 101) responsibility, only seven parents did not have full custody of their child.

All parents were invited to participate again at time 2 and 184 parents (111 mothers and 73 fathers) of 116 children (61 boys, 55 girls) participated. For 68 children, both parents participated at time 2. The study began 5 days prior to the UK’s Covid-19 lockdown in 2020, which means that all parents completed the time 2 measure during lockdown. This was not a planned part of the study design. There was no evidence that parents who completed the time 2 survey differed significantly from those who did not on parent age, parent sex, child age, child sex, primary caregiver status, number of children, parental marital status, parental employment status or parental level of education (all *ps* > .05).

### Materials

#### Children’s play scale (CPS)

##### Measure development

An initial example version of the measure was developed through informal discussion with experts in children’s play from a range of backgrounds including academic, policy and playwork, and by reviewing other relevant measures of children’s time use, physical activity and play. The main measure asked: 1) how often children play in each of a range of places; 2) how long children play for when they are in each place. In addition, the adventurous play supplement asked how adventurously children play in each place. We then held two parent consultation meetings where we invited parents to provide feedback on the draft version. During these meetings, parents first completed the draft measure and then took part in a group discussion which focused on: 1) their understanding of the questions; 2) the range of play places included and whether the list was well-defined and comprehensive; 3) their ability to answer the questions reliably and confidently; 4) any other points of feedback. The meetings lasted approximately 90 min each.

Parents for the consultation meetings were recruited via an advertisement on local social media groups. Parents were asked to register their interest and provide some basic demographic information. A total of 27 parents responded (25 mothers, 2 fathers), 14 of these were invited to join one of the two groups. Parents were selected to maximise the diversity of the groups (parent age, child age, education, ethnicity, location). Thirteen parents participated in one of the two groups. They were paid £20 for their time.

Following the first meeting, substantial changes were made to the measure and supplement and parents at the second meeting provided feedback on the revised version. Key changes that were implemented following feedback from these consultation meetings were as follows: a) parents had difficulty distinguishing between different types of playgrounds that were originally listed e.g. adventure playground, fixed playground and natural playground, so these were collapsed into a single ‘playground’ category; b) parents found it difficult to reliably separate play in wooded areas and play in open green space as many spaces had both open space and areas of woodland so these were collapsed into a single category; c) parents reported the considerable difference in play between seasons so we revised the measure to separate autumn/winter and spring/summer; d) parents wanted clarity on whether screen time should be counted as play and whether play when children were on holiday should be included or not, as holidays provide a unique opportunity for play that may be inconsistent with measures of play outside of holiday-time and therefore difficult to estimate. As a result, we chose to exclude screen time and time on holiday from the survey in the instructions. There was some discussion around the layout of the questions but on balance it was decided to keep all of the frequency questions together, followed by the time spent playing questions and then the adventurous play questions.

A final version of the measure and supplement were created based on input from both consultation groups. These were then sent via email to eleven experts on children’s play. The aim of consulting experts was to support the content validity and practical utility of the measure. The experts were members of the Children’s Play Policy Forum or Play Safety Forum in the UK who had expressed an interest in supporting the research along with two international academics who have published extensively on children’s play and adventurous play. Feedback was received via email and annotations on the survey from six individuals: the lead of a national play policy organisation who has a playwork background; an educational psychologist working in play policy; the Chief Executive Officer of an organisation who work to improve play in schools; a qualified teacher and landscape architect who leads education and community projects focused on play and outdoor learning; and the two international academics.

This feedback led to a number of changes including: a change in the wording of the response options such that they all reflected a specified period of time (the initial survey included some time periods of some options such as ‘occasionally’); the inclusion of definitions of the levels of adventurous play prior to the questions; an edit to the definition of adventurous play from ‘usually includes’ to ‘can include’; an edit to the examples of adventurous play; the addition of the option ‘Possibly but I don’t know where because they are unsupervised’ as a response to the question regarding other places children play; the inclusion of the word ‘challenge’ within the definitions of adventurous play. The experts gave some feedback and suggestions that did not result in changes, primarily because the parent feedback suggested the changes were not required. For example, one expert expressed a concern about the length, but the parent feedback was that the length was ok, one suggested we define adventurous play as courageous play, but we were confident parents understood adventurous play, and one raised a question about whether weekdays and weekends should be separated. This latter issue is something that we discussed at length in the parent sessions. Parents stated, as might be expected, that their child played more at the weekends than on weekdays but they also felt they were able to average over the full week in their responses. Importantly, they felt this was preferable to having the answer all of the questions twice, once for weekdays and once for weekends. Across both groups they felt that the seasonal differences would be harder to average across and therefore that these should be separated out rather than days of the week.

##### Description of final measure

The final measure evaluated in the present paper (available in supplementary material as Additional file 1 and for download here: https://osf.io/637rd/?view_only=e11a2e1accd843c59cecb3a54fc7767e)^1^, asked parents about their child’s play in seven places:

1. at home or in other people’s homes
2. outside at home or at other people’s homes (e.g. garden/yard/balcony)
3. at a playground
4. in trees/forests/woodland/grassy spaces (not including the garden at home or other people’s homes). This category will now be referred to as green spaces
5. in the street or public place close to home. Now referred to as street
6. outdoors near water
7. indoor play centres and pools (e.g. soft play, trampoline parks, swimming pools etc.).

The first question asked parents to report how frequently their child played in each place during Autumn/Winter. Parents responded using a seven-point scale from ‘every day’ to ‘never’, with each option anchored to a period of time. The second question asked parents to report how long their child played for in each place on a day when they played there during Autumn/Winter. For this question parents responded using a 4-point Likert scale from ‘less than half an hour’ to ‘4 hours +’. A ‘not applicable’ option was also included for any places where the child did not play. The following two questions were identical but asked parents to report about their child’s play during Spring/Summer. The fifth question formed the supplementary section on adventurous play and asked parents to rate how adventurously their child played in each place. Parents were given a definition of adventurous play (see Additional file 1) and asked to use a 5-point scale from ‘very low levels of adventure’ to ‘maximum levels of adventure’ (see definitions provided to parents in Table 1).

**Table 1:** Adventurous play levels

Very low levels of adventure [1]	Play might be fun but levels of excitement, challenge and risk are low
Mild levels of adventure [2]	Some excitement but rarely feels any fear/thrill or takes any significant challenge and risk
Moderate levels of adventure [3]	Excitement with some fear/thrill and some minor challenge and risk-taking
High levels of adventure [4]	Excitement with clear fear/thrill, challenge and risk-taking
Maximum levels of adventure [5]	Very exciting with lots of thrilling emotions and fear and obvious challenge and risk

At the end of the measure parents were asked whether there were any other places that their child played. If they answered yes to this question they were asked to state where and to answer additional questions about frequency, length of time playing and level of adventurous play in that place. Up to three additional places could be included. Definitions of each level of adventure were provided. At the beginning of the questions, parents were provided with a definition of play and were asked to think about play in children’s day to day lives, not whilst on family holidays and only outside of school, childcare and organised sports. They were also asked not to include screen time.

##### Scoring

To score the measure, first the play frequency scores were converted into approximate days (for example, ‘every day’ was converted into 182.5 days (half the year to correspond to Autumn/Winter or Spring/Summer). Where a range was selected, the centre of that range was used (e.g. 4–6 times a week was converted into 130 (5 days × 26 weeks). The length of play scores were similarly converted but into the corresponding number of hours (e.g. 2–3 h is converted into 2.5 h). The exact mappings used are shown in supplemental material (Additional file 3). The play frequency variable was then multiplied by the length of play variable for each place to give an approximate number of hours that each child played in each place. These were calculated separately for Autumn/Winter and Spring/Summer and then added together to give an estimation of total hours spent playing across the year in each place. The level of adventure scores were coded 1–5, with higher scores indicated higher levels of adventurous play. This data was then used to create four variables: total hours spent playing (created by summing across all places); total hours spent playing outdoors (created by summing across outdoor places only); total hours spent playing in nature (created by summing across natural places only: green spaces and near water); total hours spent playing in adventurous places (created by summing across the places where children were rated as playing most adventurously; playground, green spaces, indoor play centres, near water - see Fig. 2).

For the supplement, to calculate the amount of time each child spent playing adventurously, the total hours spent playing in each place were summed only across places where that child was rated as playing with an adventure rating of 2 (mild level of adventure) or more. To capture how adventurously each child played typically, the mean score across adventurous play ratings was used.

### Procedure

The study advert directed parents to a link which included the study information sheet and consent form. After completing the consent form, participants were given immediate access to the survey. All study materials were presented via SurveyMonkey. Responses were checked to ensure participants had completed the survey correctly and participants were then sent a voucher for £10 to compensate them for their time. A link to the follow-up survey was sent to participants via email 13 days after they had completed the initial survey. This directed participants to the survey, which was presented via SurveyMonkey. Participants were sent a further voucher for £10 to compensate them for their time. A reminder email and text message were sent to participants who did not respond within a week. The time between Time 1 and Time 2 ranged from 13 days to 43 days, mean = 20 days, SD = 7 days.

## Results

Data were prepared and analysed in R Studio version 1.3.1056 [20] and R version 4.0.2. The analysis script and raw data are available here: https://osf.io/637rd/?view_only=e11a2e1accd843c59cecb3a54fc7767e .

### Descriptives based on time 1 data

**Fig. 1:**
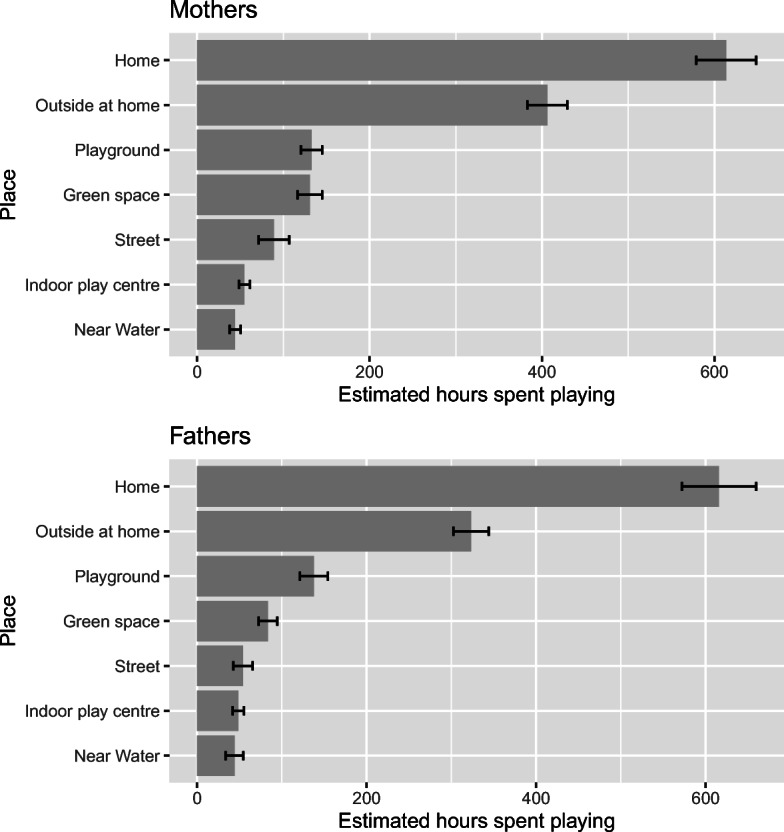
Mean hours children spent playing in each place based on mother and father report

Initially descriptive data are presented using Time 1 data for time spent playing across place and adventurous play rating across place. Figure 1 shows the mean hours spent playing in each place, as estimated based on mother and father report. Children were reported to spend more time playing inside at home or other people’s homes than in any other place. This was followed by playing outside at home and at other people’s homes. Beyond play at home, children spent most time playing at playgrounds and in green spaces. The least number of hours were spent playing at indoor play centres and near water. Mother’s and Father’s report was very similar although mothers estimated that children spent more time playing in green spaces and on the street relative to other places, than fathers. Full statistical analysis of differences across place are shown in supplementary file 2.

**Fig. 2:**
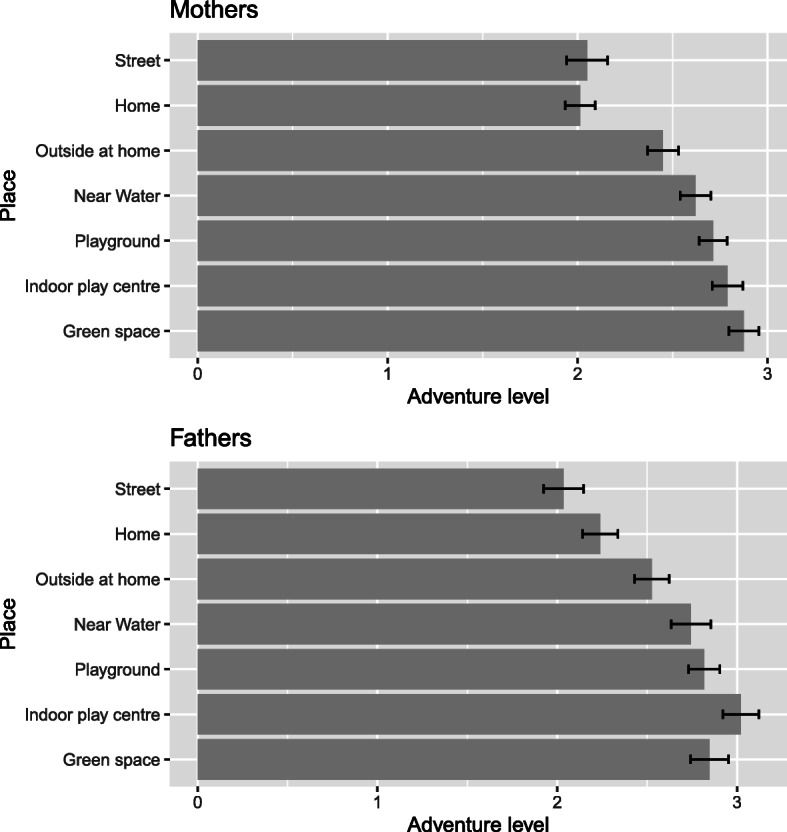
Mean level of adventure in each place based on mother and father report

Figure 2 shows the mean adventure level for play in each place. This indicates that play was least adventurous at home and on the street near home. The adventurousness of play was slightly greater for play outdoors at home or other people’s homes and again, slightly more adventurous near water and on playgrounds. Play was most adventurous at indoor play centres and in green spaces, although Mothers and Fathers disagreed on which of these was the most adventurous place. Full statistical analysis of differences in adventurous play ratings across place, including examination of child gender differences (no significant gender differences were found for any metric), are shown in supplementary material as Additional file 2.

### Stability from time 1 to time 2

Table 2 shows the means and standard deviations for each variable at time 1 and time 2 for mothers and fathers. Paired sample t-tests indicated that there were no significant differences between mothers and fathers on any of the variables (all *p’s* > .05). Furthermore, paired t-tests showed that most of the scores from time 1 to time 2 were stable and did not differ significantly. The only exception was ratings of average level of adventurous play, which were higher at time 2.

**Table 2:** Mother and Father means and standard deviations for each metric at Time 1 and Time 2

	T1		T2	
	Mothers	Fathers	Mothers	Fathers
1. Total hours spent playing	1470.36 (864.57)	1296.30 (615.39)	1346.99 (720.52)	1273.45 (733.17)
2. Hours spent playing outdoors	799.27 (610.43)	631.96 (356.90)	717.58 (496.23)	659.82 (472.40)
3. Hours spent playing in nature	175.58 (215.45)	127.24 (171.87)	173.36 (214.88)	136.66 (196.76)
4. Hours spent playing in adventurous places	364.39 (359.05)	304.02 (253.43)	345.19 (345.01)	306.27 (313.95)
5. Hours spent playing adventurously	1176.9 (916.33)	1101.62 (676.51)	1227.11 (758.9)	1173.15 (783.05)
6. Average level of adventurous play	2.53 (0.76)	2.62 (0.72)	2.74^**^ (0.61)	2.72^*^ (0.56)

Note. Time 1 to Time 2 change is significant at * *p* < .05; ** *p* < .001

### Reliability

Concordance Correlation Coefficients (CCCs) were computed using the CCC function in epiR to assess the agreement between caregivers of the same child on each of the metrics of interest at time 1 and also to assess test-retest reliability for mothers and fathers separately across time 1 and time 2. CCCs were used over Intraclass Correlation Coefficients because many of the variables were not normally distributed.

#### Cross-informant agreement

As shown in Table 3, for the majority of metrics, there was poor to moderate agreement between caregivers based on time 1 data.

**Table 3:** Concordance Correlation Coefficients (CCC) for cross-informant and test-retest reliability for each metric

	Cross-informant agreement CCC [upper and lower bounds]	Test retest reliability CCC Mothers [upper and lower bounds]	Test retest reliability CCC Fathers [upper and lower bounds]
1. Total hours spent playing	0.51 [0.34–0.65]	0.73 [0.62–0.80]	0.47 [0.26–0.64]
2. Hours spent playing outdoors	0.49 [0.32–0.63]	0.68 [0.56–0.77]	0.49 [0.29–0.65]
3. Hours spent playing in nature	0.36 [0.17–0.52]	0.73 [0.63–0.80]	0.61 [0.45–0.74]
4. Hours spent playing in adventurous places	0.44 [0.26–0.59]	0.76 [0.67–0.83]	0.63 [0.47–0.76]
5. Hours spent playing adventurously	0.37 [0.18–0.54]	0.67 [0.55–0.76]	0.39 [0.16–0.57]
6. Average level of adventurous play	0.41 [0.22–0.56]	0.49 [0.35–0.61]	0.42 [0.22–0.58]

#### Test-rest reliability

For the majority of metrics, the reliability fell in the moderate range, although there are notable exceptions. Father report of total hours spent playing, hours spent playing outdoors and hours spent playing adventurously did not reach the threshold for moderate reliability and were substantially lower than the reliability of the same metrics for mothers. Additional analyses were conducted to examine whether reliability differed by primary caregiver status (see supplementary file 2 for results). As expected, primary caregivers were typically more reliable over time than non-primary caregivers. Further, for both parents, reliability was notably lower for both parents for the average level of adventurous play variable, relative to the other metrics. This may be due to the timing of the survey in relation to the Covid-19 pandemic and adjustments in risk perception, which is consistent with the Time 1 to Time 2 change, shown in Table 1.

#### Internal consistency

For each of the time variables, the estimated time spent playing in each place is summed to create the total. Given this, we would not expect there to be strong internal consistency across items; the more time a child spends playing in one place decreases the available time for them to play in another place. Consistent with this, the Cronbach’s alpha, using time 1 data, ranged from 0.32 to 0.66 for these variables. In contrast, the mean level of adventurous play rating is calculated based on parent’s ratings of how adventurously their child plays across places. Whilst we would expect higher levels of adventurous play in some places relative to others, we might also expect some consistency within each child. This is reflected in the internal consistency for this variable which, based on time 1 data, was good (Mothers α = 0.89; Fathers α = 0.89).

#### ‘Other’ responses

Fifteen percent of participants reported that there was another place that their child played in an adventurous way. Only 17 participants (< 4%) stated that their child may play in other places but they do so unsupervised. Table 4 shows the frequency with which other places were identified as places where children might play adventurously.

**Table 4:** Categories of places listed under ‘other places your child plays adventurously’ and number (proportion) of participants listing each place

Place	Number (percentage) of parents listing ‘other’ place
School	10 (4.1%)
Theme parks/Adventure parks	9 (3.7%)
Cycling/Mountain biking	7 (2.9%)
Climbing Walls	5 (2.0%)
Farms/allotments	4 (1.6%)
Go Ape rope courses	4 (1.6%)
Beavers and Cub Scouts	3 (1.2%)

Others that were only mentioned once were: ski slope, skate park, cricket club, camping, forest school, gymnastics, DIY. Thirteen participants listed places that had been included in the CPS (at home, swimming, woods, other people’s gardens etc.).

## Discussion

This article describes the development of the Children’s Play Scale (CPS), a parent-report questionnaire that could be used to quantify school-aged children’s time spent playing in a range of places and their engagement in adventurous play. The measure was designed with input and feedback from a diverse group of parents as well as experts on children’s play who came from a range of perspectives (academic, play work, play policy, psychology). The reliability of the measure was evaluated in two ways. First, via cross-informant reliability where two parents completed the measure about the same child. Second, via test-retest reliability where the same parent completed the measure twice approximately 3 weeks apart.

By including mothers and fathers of the same children we were able to evaluate cross-informant reliability. It is common for parent agreement to be poor to moderate for measures that ask about their children [21]. For the CPS metrics, all fell within the poor to moderate range. To give some context, the CCCs were comparable to the cross-informant correlation coefficients reported for well-validated measures regularly included in large scale public health research, including the subscales of the Strengths and Difficulties Questionnaire [22], the Social Skills Rating System and the Conner’s Rating Scale [23].

Because both parents reported on the same child, test-retest reliability was calculated for mothers and fathers separately. Overall, test-retest reliability was higher for mothers than for fathers. Reliability also tended to be higher for parents who identified as primary caregiver than those who did not. Given that mothers are more likely to be primary caregivers and to spend more time with their children [24], these results are consistent. It is interesting that the reliability for fathers was stronger for play in nature and play in adventurous places, relative to the other metrics. This may be because father’s play with their children is more likely to take place in nature or adventurous places, allowing them to be more accurate in their estimations. This would be consistent with theory about father-child play (e.g. [25]) but we did not collect data in the present study about differences in play location or time spent playing between parents.

We spent some time during the development of the measure refining the list of places where children might play. Having begun with a longer list of more specific places, we collapsed across some places following feedback from parents that they weren’t able to reliably separate them. We wanted to keep the list relatively short for practical reasons but we recognised that children may occasionally play in other places. To check how well we had captured the places where children play, we asked participants whether there were any other places that their child played. A small minority listed other places but there was little consistency in what these places were and there is some question over whether the activities in these places should be considered play (e.g. theme parks). Taken together, the findings support the use of the measure and indicate that the list of places is broad enough to capture most of the places where UK children play.

Initial results from the measure showed that children spent more time playing at home or in other people’s homes than anywhere else. This was followed by play outside at home or other people’s homes. Away from home children were most likely to play at the park or in green spaces. These results align with research using accelerometers and place-logs, which showed that children spent almost 50% of their time at home [26]. Other than at home or school, Perry and colleagues found that children primarily spent time at other people’s homes or in parks, which also aligns with our findings. Interestingly, in relation to children’s play and physical activity, Perry et al. found that children were most physically active when they were in their neighbourhood or in parks. In terms of adventurous play, parents reported that children played most adventurously at playgrounds, in green spaces and at indoor play centres, which included soft play, trampoline parks and swimming pools. They played least adventurously at home. These findings provide a good example of how the measure might be utilised in future research to provide insights into children’s activity and use of space.

The measure yields a range of metrics that could be used in future research, according to need. For example, research focused on children’s play in nature may only require the time spent playing nature metric. Similarly, research focused on outdoor play specifically might use the outdoor play metric only. The adventurous play supplement can be included or not, as required. As well as yielding metrics to examine different places children play, the measure allows for flexibility and could also be used to examine seasonal differences in the amount of time children spend playing. We made the decision not to separate out weekday play from weekend play following feedback from parents that they didn’t want the measure to be any longer and they felt able to average across the days of the week. This necessarily means that the measure can’t be used to differentiate between play that happens during the week and play that happens at the weekend. If required for future research, the measure could be adapted to separate these out, although ideally this would need to evaluated as a new version of the measure. From a public health perspective, the measure could be used to evaluate associations between children’s outdoor play and physical health for example as well as between their adventurous play and mental health. The measure could also be used alongside Geographic Information System (GIS) data to evaluate how the space planning and access to green space affects children’s outdoor play and/or play in nature. There is also scope for using the measure to examine cross-cultural differences in other countries, although this will require further validation work to ensure that the play places are relevant and that the measure is reliable in other countries and cultures. Future work may also benefit from examining how the reliability of the measure may differ depending on child age. Unfortunately, this study was not sufficiently powered to examine these differences, it may be expected that parents of younger children would be more reliable in reporting on their child’s play, and that as children age and have greater independence, parents may be less reliable in monitoring their child’s play activities, this warrants further investigation.

The current study has a number of strengths including the careful development of the measure with input from parents and experts and the inclusion of both mothers and fathers. We evaluated both test-retest reliability as well as cross-informant reliability. A significant limitation is that the measure was not evaluated against more objective measures such as accelerometers, observation or time use diaries. Validating the survey in this way is challenging because there is no gold-standard objective measure of children’s play; each of the available options has its own weaknesses. For example, an accelerometer would give information on physical activity but not all play involves physical activity and not all physical activity is playful. Similarly, a time-use diary places a heavy burden on participants and provides only a snapshot of children’s play within a short period. Nevertheless, we would expect that the metrics should correlate somewhat with physical activity and a time-use diary and future research evaluating the validity of the current measure against these alternatives would provide an important further evaluation of the measure. In relation to the level of adventurous play, no objective measures exist of children’s risk-taking in play to validate the measure against, but the measure might be useful in research designed to develop observational measures of children’s risk-taking in play. Although it wasn’t possible to evaluate the convergent validity of the measure against other measures, the face validity of the measure is strong given the collaborative process of measure development taken.

A further limitation is that the average level of adventurous play rating reliability was relatively low. A possible, albeit posthoc, explanation for this is that the baseline of the survey began 5 days before the Covid-19 UK-wide lockdown, with all participants completing follow-up during lockdown. It seems likely that during this unusual time, parent’s perception of risk-taking and adventure for their child may have shifted somewhat, depending on their own personal circumstances and their children’s access to adventurous play during lockdown. In keeping with this, previous research has shown that perceptions of risk may shift in light of traumatic or unprecedented events [27, 28]. Furthermore, this variable was the only variable to show significant change over time, with parents reporting at time 2, during the Covid-19 lockdown, that the way their child typically played pre-lockdown was more adventurous than they had perceived it to be at time 1. The timings are unfortunate and may explain the slightly lower reliability estimates for average level of adventurous play. Until the risks associated with Covid-19 are diminished it will not be possible to evaluate this explanation but it would be beneficial to evaluate the test-retest reliability of the measure again once restrictions related to Covid-19 are lifted. The present research provides a strong foundation for the further development and evaluation of this measure. The next steps for building on this work are to replicate the current findings, including test-retest reliability, during a more stable time, to examine validity against physical activity trackers and time use diaries, to collect data on a larger sample who are more representative of the general population and to examine the use of the measure in other cultures.

## Conclusion

In conclusion, we have presented a new measure for capturing children’s time spent playing in a range of places, via parent report. The measure extends previous work in a number of ways, including by adding ratings of children’s adventurous play in each place. Overall, a number of the metrics that the measure yields have adequate reliability, especially based on maternal report. The measure will be useful in a range of future public health research.

## Supplementary Information

**Additional file 1.** Questionnaire as used in this paper.

**Additional file 2.** Further analyses.

**Additional file 3.** Numeric mappings.

## Data Availability

The dataset generated and analysed during the current study and the analysis script are available in the Open Science Framework repository via the following link: https://osf.io/637rd/?view_only=e11a2e1accd843c59cecb3a54fc7767e.

## Footnotes

1 Note that the data collection for this study began 5 days prior to the Covid-19 UK-wide lockdown. Because of this we included instructions for participants to answer all of the questions in the survey ‘thinking about normal life before coronavirus’. These additions can be seen in the version of the measure shown here: https://osf.io/637rd/?view_only=e11a2e1accd843c59cecb3a54fc7767e but would not be necessary for versions of the measure used in future research.
