# Supplementary material for: Development and evaluation of a new measure of children’s play: the Children’s Play Scale (CPS)
Source: BMC Public Health. 2021 May 7;21:878. doi: 10.1186/s12889-021-10812-x (PMC8103430; doi:10.1186/s12889-021-10812-x)
Supplement: Supplementary file 1 — Additional file 1. Questionnaire as used in this paper. [file 12889_2021_10812_MOESM1_ESM.pdf]

Children's Spare Time survey - Part 1

The next questions ask about where your child plays at different times of year, how long they play for in each place and how adventurously they play.

Please answer all of the questions in this survey thinking about normal life before coronavirus!

Most of the questions ask about children's play generally.

Play is any activity that a child does for enjoyment where there is no serious or practical purpose. Lessons and sports training for example do not count as play here but playing with a ball with friends does.

We would like you to think about play in children's day to day lives, not whilst on family holidays and only outside of school, childcare and organised sports.

26. Please select how often your child typically plays in each of the following places <u>during Autumn and Winter.</u>

You should respond thinking about day-to-day life, not whilst on holiday away from home and do not include screen time.

	Every day	4-6 times per week	2-3 times per week	Once a week	Once a month	Less than once a month	Never
At home or in other people's homes	○	○	○	○	○	○	○
Outside at home or at other people's homes (e.g. garden/yard/balcony)	○	○	○	○	○	○	○
At a playground	○	○	○	○	○	○	○
In trees/forests/woodland/grassy spaces (not including the garden at home or other people's homes)	○	○	○	○	○	○	○
In the street or public space close to your home (e.g. in your street, on the pavement in front of your home etc.) (not including local playgrounds)	○	○	○	○	○	○	○
Outdoors near water (e.g. at the beach, in the sea, near a river, lake or cliffs)	○	○	○	○	○	○	○
At indoor play centres and pools (e.g. soft play, trampoline parks, swimming pools etc.)	○	○	○	○	○	○	○

27. On a day when your child does play in a place listed, please select how long your child typically plays for in each place during Autumn and Winter.

If they never play in a particular place select N/A.

You should respond thinking about day-to-day life, not whilst on holiday away from home and do not include screen time.

	Less than half an hour	Around an hour	2-3 hours	4 hours+	N/A
At home or in other people's homes	○	○	○	○	○
Outside at home or at other people's homes (e.g. garden/yard/balcony)	○	○	○	○	○
At a playground	○	○	○	○	○
In trees/forests/woodland/grassy spaces (not including the garden at home or other people's homes)	○	○	○	○	○
In the street or public space close to your home (e.g. in your street, on the pavement in front of your home etc.) (not including local playgrounds)	○	○	○	○	○
Outdoors near water (e.g. at the beach, in the sea, near a river, lake or cliffs)	○	○	○	○	○
At indoor play centres and pools (e.g. soft play, trampoline parks, swimming pools etc.)	○	○	○	○	○

Children's Spare Time survey - Part 1

Spring/Summer activities

Remember to think about normal life before coronavirus!

28. Please select how often your child typically plays in each of the following places during Spring and Summer.

You should respond thinking about day-to-day life, not whilst on holiday away from home and do not include screen time.

	Every day	4-6 times per week	2-3 times per week	Once a week	Once a month	Less than once a month	Never
At home or in other people's homes	○	○	○	○	○	○	○
Outside at home or at other people's homes (e.g. garden/yard/balcony)	○	○	○	○	○	○	○
At a playground	○	○	○	○	○	○	○
In trees/forests/woodland/grassy spaces (not including the garden at home or other people's homes)	○	○	○	○	○	○	○
In the street or public space close to your home (e.g. in your street, on the pavement in front of your home etc.) (not including local playgrounds)	○	○	○	○	○	○	○
Outdoors near water (e.g. at the beach, in the sea, near a river, lake or cliffs)	○	○	○	○	○	○	○
At indoor play centres and pools (e.g. soft play, trampoline parks, swimming pools etc.)	○	○	○	○	○	○	○

29. On a day when your child does play in a place listed, please select how long your child typically plays for in each place during Spring and Summer.

If they never play in a particular place select N/A.

You should respond thinking about day-to-day life, not whilst on holiday away from home and do not include screen time.

	Less than half an hour	Around an hour	2-3 hours	4 hours+	N/A
At home or in other people's homes	◯	◯	◯	◯	◯
Outside at home or at other people's homes (e.g. garden/yard/balcony)	◯	◯	◯	◯	◯
At a playground	◯	◯	◯	◯	◯
In trees/forests/woodland/grassy spaces (not including the garden at home or other people's homes)	◯	◯	◯	◯	◯
In the street or public space close to your home (e.g. in your street, on the pavement in front of your home etc.) (not including local playgrounds)	◯	◯	◯	◯	◯
Outdoors near water (e.g. at the beach, in the sea, near a river, lake or cliffs)	◯	◯	◯	◯	◯
At indoor play centres and pools (e.g. soft play, trampoline parks, swimming pools etc.)	◯	◯	◯	◯	◯

Children's Spare Time survey - Part 1

Adventurous play

Adventurous play is play that is thrilling and exciting where your child might experience some fear and uncertainty.

Adventurous play can include playing high up, moving so fast they feel that they might lose control, playing near open water or a cliff, play fighting, playing with tools that could be dangerous or playing somewhere they could get lost and are out of sight of adults. For example, jumping off something very high, climbing high, riding a bike very fast, exploring woodland alone or with a friend, would all be examples of adventurous play.

You will be asked how adventurously your child plays at each place, please indicate your response using the five options below.

Very low levels of adventure – Play might be fun but levels of excitement, challenge and risk are low.
Mild levels of adventure – Some excitement but rarely feels any fear/thrill or takes any significant challenge and risk.
Moderate levels of adventure – Excitement with some fear/thrill and some minor challenge and risk-taking.
High levels of adventure – Excitement with clear fear/thrill, challenge and risk-taking.
Maximum levels of adventure – Very exciting with lots of thrilling emotions and fear and obvious challenge and risk.

30. Now select how adventurously your child plays when they play in each of these places.

If they never play in a particular place please select N/A.

You should respond thinking about day-to-day life, not whilst on holiday away from home and do not include screen time.

	Very low levels of adventure	Mild levels of adventure	Moderate levels of adventure	High levels of adventure	Maximum level of adventure	N/A
At home or in other people's homes	○	○	○	○	○	○
Outside at home or at other people's homes (e.g. garden/yard/balcony)	○	○	○	○	○	○
At a playground	○	○	○	○	○	○
In trees/forests/woodland/grassy spaces (not including the garden at home or other people's homes)	○	○	○	○	○	○
In the street or public space close to your home (e.g. in your street, on the pavement in front of your home etc.) (not including local playgrounds)	○	○	○	○	○	○
Outdoors near water (e.g. at the beach, in the sea, near a river, lake or cliffs)	○	○	○	○	○	○
At indoor play centres and pools (e.g. soft play, trampoline parks, swimming pools etc.)	○	○	○	○	○	○

31. Is there another place where your child regularly plays in an adventurous way?

○ No

○ Possibly but I don't know where because they are unsupervised

○ Yes (please specify)

[ ]

University of Reading

Children's Spare Time survey - Part 1

Other

32. How often does your child play in this place?

	Never/Hardly ever	Occasionally	Once or twice a month	Once a week	A couple of times a week	Every day
During Autumn/Winter	○	○	○	○	○	○
During Spring/Summer	○	○	○	○	○	○

33. On a day when your child does play in this place, how long does your child get to spend on average playing?

	Less than half an hour	Around an hour	2-3 hours	4+ hours
During Autumn/Winter	○	○	○	○
During Spring/Summer	○	○	○	○

34. How adventurously does your child play when they play here?

Very low levels of adventure	Mild levels of adventure	Moderate levels of adventure	High levels of adventure	Maximum levels of adventure
○	○	○	○	○

University of Reading

Children's Spare Time survey - Part 1

Other

35. Is there another place where your child regularly plays in an adventurous way?

- No
- Possibly but I don't know where because they are unsupervised
- Yes (please specify)

Children's Spare Time survey - Part 1

Other

36. How often does your child play in this place?

	Never/Hardly ever	Occasionally	Once or twice a month	Once a week	A couple of times a week	Every day
During Autumn/Winter	◯	◯	◯	◯	◯	◯
During Spring/Summer	◯	◯	◯	◯	◯	◯

37. On a day when your child does play in this place, how long does your child get to spend on average playing?

	Less than half an hour	Around an hour	2-3 hours	4+ hours
During Autumn/Winter	◯	◯	◯	◯
During Spring/Summer	◯	◯	◯	◯

38. How adventurously does your child play when they play here? (select n/a if they do not play here).

Very low levels of adventure	Mild levels of adventure	Moderate levels of adventure	High levels of adventure	Maximum levels of adventure
◯	◯	◯	◯	◯

University of Reading

Children's Spare Time survey - Part 1

Other

39. Is there another place where your child regularly plays in an adventurous way?

- ◯ No
- ◯ Possibly but I don't know where because they are unsupervised
- ◯ Yes (please specify)

Children's Spare Time survey - Part 1

Other

40. How often does your child play in this place?

	Never/Hardly ever	Occasionally	Once or twice a month	Once a week	A couple of times a week	Every day
During Autumn/Winter	○	○	○	○	○	○
During Spring/Summer	○	○	○	○	○	○

41. On a day when your child does play in this place, how long does your child get to spend on average playing?

	Less than half an hour	Around an hour	2-3 hours	4+ hours
During Autumn/Winter	○	○	○	○
During Spring/Summer	○	○	○	○

42. How adventurously does your child play when they play here?

Very low levels of adventure	Mild levels of adventure	Moderate levels of adventure	High levels of adventure	Maximum levels of adventure
○	○	○	○	○
