# Supplementary material for: Development and evaluation of a new measure of children’s play: the Children’s Play Scale (CPS)
Source: BMC Public Health. 2021 May 7;21:878. doi: 10.1186/s12889-021-10812-x (PMC8103430; doi:10.1186/s12889-021-10812-x)
Supplement: Supplementary file 2 — Additional file 2. Further analyses. [file 12889_2021_10812_MOESM2_ESM.docx]

**Supplemental Material**

**Analysis of time spent playing across place**

A one-way ANOVA indicated that the amount of time children spent playing differed significantly by place for both mother, *F* (2.69, 384.47) = 151.12, *p* < .001, and father, *F* (1.93, 173.51) = 112.06, *p* < .001, report (see Figure 1). Bonferroni-corrected pairwise comparisons were conducted to compare time spent playing in each place for mothers and father separately. The results of these pairwise comparisons are shown in table S1 and S2 below.

**Analysis of level of adventurous play across place**

A one-way ANOVA showed that the level of adventure in play varied significantly across place for both mother report, F (4.84, 324) = 19.47, *p* < .001, and father report, F (4.71, 221.28) = 13.65, *p* < .001 (see Figure 2). Bonferroni adjusted pairwise comparisons are shown in Table S3 and S4 below.

Table S1.

*Bonferroni-corrected pairwise comparisons comparing time spent playing in each place for mothers.*

| **Place 1** | **Place 2** | **Statistic** | ***df*** | ***p-value*** | ***Adjusted p-value*** |
| --- | --- | --- | --- | --- | --- |
| Home play | Outside home play | 6.45 | 146 | 1.57e-09 | 3.30e-08^****^ |
| Home play | Playground play | 13.11 | 144 | 2.51e-26 | 5.27e-27^****^ |
| Home play | Green space | 13.47 | 146 | 2.18e-27 | 4.58e-26^****^ |
| Home play | Street play | 13.96 | 146 | 1.13e-28 | 2.37e-27^****^ |
| Home play | Indoor centre play | 16.04 | 146 | 4.99e-34 | 1.05e-32^****^ |
| Home play | Near water play | 16.64 | 145 | 1.97e-35 | 4.14e-35^****^ |
| Outside home play | Playground play | 11.86 | 146 | 3.78e-23 | 7.94e-22^****^ |
| Outside home play | Green space | 11.84 | 148 | 3.58e-23 | 7.52e-22^****^ |
| Outside home play | Street play | 13.15 | 148 | 1.22e-26 | 2.56e-25^****^ |
| Outside home play | Indoor centre play | 14.80 | 148 | 5.39e-31 | 1.13e-29^****^ |
| Outside home play | Near water play | 18.89 | 147 | 1.02e-33 | 2.14e-32^****^ |
| Playground play | Green space | 0.08 | 146 | 9.36e-01 | 1.00e+00*^ns^* |
| Playground play | Street play | 2.16 | 146 | 3.20e-02 | 6.80e-01*^ns^* |
| Playground play | Indoor centre play | 6.21 | 146 | 5.23e-09 | 1.10e-07^****^ |
| Playground play | Near water play | 7.42 | 145 | 9.28e-12 | 1.95e-10^****^ |
| Green space | Street play | 2.17 | 148 | 3.20e-02 | 6.62e-01*^ns^* |
| Green space | Indoor centre play | 5.30 | 148 | 4.05e-07 | 8.50e-06^****^ |
| Green space | Near water play | 6.73 | 147 | 3.60e-10 | 7.56e-09^****^ |
| Street play | Indoor centre play | 1.80 | 148 | 7.30e-02 | 1.00e+00*^ns^* |
| Street play | Near water play | 2.31 | 147 | 2.20e-02 | 4.70e-01*^ns^* |
| Indoor centre play | Near water play | 1.50 | 147 | 1.35e-01 | 1.00e+00*^ns^* |

Note. *ns* = not significant; ^*^ < .05; ^**^ < .01; ^***^<.001; ^****^<.0001.

Table S2.

*Bonferroni-corrected pairwise comparisons comparing time spent playing in each place for fathers.*

| **Place 1** | **Place 2** | **Statistic** | ***df*** | ***p-value*** | ***Adjusted p-value*** |
| --- | --- | --- | --- | --- | --- |
| Home play | Outside home play | 7.50 | 92 | 3.92e-11 | 8.23e-10^****^ |
| Home play | Playground play | 10.06 | 92 | 1.74e-16 | 3.65e-15^****^ |
| Home play | Green space | 11.58 | 92 | 1.18e-19 | 2.48e-18^****^ |
| Home play | Street play | 12.73 | 92 | 5.39e-22 | 1.13e-20^****^ |
| Home play | Indoor centre play | 12.85 | 91 | 3.76e-22 | 7.90e-21^****^ |
| Home play | Near water play | 12.25 | 91 | 5.87e-21 | 1.23e-19^****^ |
| Outside home play | Playground play | 7.15 | 92 | 1.98e-10 | 4.16e-09^****^ |
| Outside home play | Green space | 10.79 | 92 | 5.13e-18 | 1.08e-16^****^ |
| Outside home play | Street play | 12.01 | 92 | 1.55e-20 | 3.26e-19^****^ |
| Outside home play | Indoor centre play | 12.69 | 91 | 7.64e-22 | 1.60e-20^****^ |
| Outside home play | Near water play | 12.09 | 91 | 1.25e-20 | 2.62e-19^****^ |
| Playground play | Green space | 3.00 | 92 | 3.00e-03 | 7.30e-02*^ns^* |
| Playground play | Street play | 4.33 | 92 | 3.74e-05 | 7.85e-04*^***^* |
| Playground play | Indoor centre play | 5.60 | 91 | 2.26e-07 | 4.75e-06^****^ |
| Playground play | Near water play | 5.03 | 91 | 2.48e-06 | 5.21e-05^****^ |
| Green space | Street play | 1.93 | 92 | 5.60e-02 | 1.00e+00*^ns^* |
| Green space | Indoor centre play | 2.76 | 91 | 7.00e-03 | 1.47e-01*^ns^* |
| Green space | Near water play | 3.22 | 91 | 2.00e-03 | 3.70e-02^*^ |
| Street play | Indoor centre play | 0.41 | 91 | 6.84e-01 | 1.00e+00*^ns^* |
| Street play | Near water play | 0.62 | 91 | 5.37e-01 | 1.00e+00*^ns^* |
| Indoor centre play | Near water play | 0.19 | 90 | 8.50e-01 | 1.00e+00*^ns^* |

Note. *ns* = not significant; ^*^ < .05; ^**^ < .01; ^***^<.001; ^****^<.0001.

Table S3.

*Bonferroni-corrected pairwise comparisons comparing level of adventurous play in each place for mothers.*

| **Place 1** | **Place 2** | **Statistic** | ***df*** | ***p-value*** | ***Adjusted p-value*** |
| --- | --- | --- | --- | --- | --- |
| Home play | Outside home play | -7.13 | 148 | 4.21e-11 | 8.84e-10^****^ |
| Home play | Playground play | -10.31 | 146 | 4.60e-19 | 9.66e-18^****^ |
| Home play | Green space | -9.98 | 143 | 3.91e-18 | 8.21e-17^****^ |
| Home play | Street play | -0.76 | 79 | 4.50e-01 | 1.00e+00*^ns^* |
| Home play | Indoor centre play | -8.97 | 141 | 1.63e-15 | 3.42e-14^****^ |
| Home play | Near water play | -6.10 | 131 | 1.09e-08 | 2.29e-07^****^ |
| Outside home play | Playground play | -3.98 | 146 | 1.07e-04 | 2.00e-03^**^ |
| Outside home play | Green space | -5.86 | 143 | 3.11e-08 | 6.53e-7^****^ |
| Outside home play | Street play | 3.41 | 79 | 1.00e-03 | 2.20e-02^*^ |
| Outside home play | Indoor centre play | -3.65 | 141 | 3.70e-04 | 8.00e-03^**^ |
| Outside home play | Near water play | -1.72 | 131 | 8.80e-02 | 1.00e+00*^ns^* |
| Playground play | Green space | -2.64 | 142 | 9.00e-03 | 1.93e-01*^ns^* |
| Playground play | Street play | 5.58 | 78 | 3.42e-07 | 7.18e-06^****^ |
| Playground play | Indoor centre play | -1.05 | 140 | 2.94e-01 | 1.00e+00*^ns^* |
| Playground play | Near water play | 1.26 | 130 | 2.10e-01 | 1.00e+00*^ns^* |
| Green space | Street play | 7.21 | 76 | 3.59e-10 | 7.54e-09^****^ |
| Green space | Indoor centre play | 0.89 | 137 | 3.72e-01 | 1.00e+00*^ns^* |
| Green space | Near water play | 3.62 | 127 | 4.25e-04 | 9.00e-03^**^ |
| Street play | Indoor centre play | -6.27 | 76 | 1.98e-08 | 4.16e-07^****^ |
| Street play | Near water play | -4.78 | 71 | 9.29e-06 | 1.95e-04^***^ |
| Indoor centre play | Near water play | 1.80 | 125 | 7.50e-02 | 1.00e+00*^ns^* |

Note. *ns* = not significant; ^*^ < .05; ^**^ < .01; ^***^<.001; ^****^<.0001.

Table S4.

*Bonferroni-corrected pairwise comparisons comparing level of adventurous play in each place for fathers.*

| **Place 1** | **Place 2** | **Statistic** | ***df*** | ***p-value*** | ***Adjusted p-value*** |
| --- | --- | --- | --- | --- | --- |
| Home play | Outside home play | -3.55 | 91 | 6.09e-04 | 1.30e-02^*^ |
| Home play | Playground play | -6.04 | 91 | 3.37e-08 | 7.08e-07^****^ |
| Home play | Green space | -5.45 | 83 | 5.13e-07 | 1.08e-05^****^ |
| Home play | Street play | 2.24 | 55 | 2.90e-02 | 6.17e-01*^ns^* |
| Home play | Indoor centre play | -8.34 | 90 | 8.01e-13 | 1.68e-11^****^ |
| Home play | Near water play | -3.52 | 77 | 7.33e-04 | 1.50e-02^*^ |
| Outside home play | Playground play | -3.49 | 92 | 7.40e-04 | 1.60e-02^*^ |
| Outside home play | Green space | -3.40 | 84 | 1.00e-03 | 2.20e-02^*^ |
| Outside home play | Street play | 5.99 | 56 | 1.58e-07 | 3.32e-06^****^ |
| Outside home play | Indoor centre play | -4.91 | 91 | 3.94e-06 | 8.27e-05^****^ |
| Outside home play | Near water play | -1.20 | 77 | 2.34e-01 | 1.00e+00*^ns^* |
| Playground play | Green space | -0.25 | 84 | 8.04e-01 | 1.00e+00*^ns^* |
| Playground play | Street play | 6.69 | 56 | 1.11e-08 | 2.33e-07^****^ |
| Playground play | Indoor centre play | -2.42 | 91 | 1.80e-02 | 3.70e-01*^ns^* |
| Playground play | Near water play | 1.04 | 77 | 3.02e-01 | 1.00e+00*^ns^* |
| Green space | Street play | 7.03 | 54 | 3.63e-09 | 7.62e-08^****^ |
| Green space | Indoor centre play | -1.92 | 83 | 5.90e-02 | 1.00e+00*^ns^* |
| Green space | Near water play | 1.80 | 74 | 7.50e-02 | 1.00e+00*^ns^* |
| Street play | Indoor centre play | -8.03 | 56 | 6.88e-11 | 1.44e-09^****^ |
| Street play | Near water play | -4.74 | 47 | 2.03e-05 | 4.26e-04^***^ |
| Indoor centre play | Near water play | 3.23 | 76 | 2.00e-03 | 3.80e-02^*^ |

Note. *ns* = not significant; ^*^ < .05; ^**^ < .01; ^***^<.001; ^****^<.0001.

Table S5.

*Mean (SD) for each metric reported by mothers and fathers, for girls and boys, respectively.*

|  | ***Mothers*** | | | | | ***Fathers*** | | | | | |
| --- | --- | --- | --- | --- | --- | --- | --- | --- | --- | --- | --- |
|  | Girls | Boys | *df* | *t* | *p* | Girls | Boys | *df* | *t* | *p* |  |
| 1. Total hours spent playing | 1424.33(846.8) | 1617.69  (886.0) | 141.24 | 0.65 | .519 | 1294.17(662.2) | 1355.94(611.3) | 90.92 | 0.47 | .641 |  |
| 2. Hours spent playing outdoors | 752.21  (618.5) | 846.64  (602.5) | 144 | 0.94 | .347 | 673.21  (386.0) | 611.86  (333.0) | 87.32 | 0.82 | .414 |  |
| 3. Hours spent playing in nature | 152.77  (199.9) | 198.39  (229.0) | 143.38 | 1.29 | .199 | 156.64  (225.0) | 104.22  (97.7) | 58.67 | 1.45 | .154 |  |
| 4. Hours spent playing in adventurous places | 340.64  (358.7) | 388.80  (360.3) | 143.85 | 0.81 | .420 | 333.42  (293.2) | 298.32  (227.3) | 80.85 | 0.64 | .524 |  |
| 5. Hours spent playing adventurously | 1038.34(905.0) | 1319.37  (912.3) | 141.82 | 1.86 | .066 | 1064.94  (638.6) | 1194.37  (754.6) | 89.45 | 0.89 | .376 |  |
| 6. Average level of adventurous play | 2.45  (0.82) | 2.61  (0.70) | 143.93 | 1.32 | .188 | 2.61  (0.68) | 2.62  (0.76) | 93.95 | 0.03 | .973 |  |

Table S6.

*Test-retest reliability for each metric for primary caregivers and non-primary caregivers, respectively.*

|  | **Test-retest reliability CCC Primary Caregivers**  **[upper and lower bounds]** | **Test-retest reliability CCC Non-Primary Caregivers**  **[upper and lower bounds]** |
| --- | --- | --- |
| 1. Total hours spent playing | 0.61  [0.47 – 0.73] | 0.67  [0.52 – 0.77] |
| 2. Hours spent playing outdoors | 0.61  [0.46 – 0.72] | 0.58  [0.42 – 0.71] |
| 3. Hours spent playing in nature | 0.70  [0.59 – 0.79] | 0.56  [0.41 – 0.68] |
| 4. Hours spent playing in adventurous places | 0.73  [0.62 – 0.81] | 0.56  [0.39 – 0.69] |
| 5. Hours spent playing adventurously | 0.60  [0.45 – 0.71] | 0.49  [0.30 – 0.64] |
| 6. Average level of adventurous play | 0.48  [0.33 – 0.60] | 0.43  [0.26 – 0.59] |
