# Supplementary material for: Development and evaluation of a new measure of children’s play: the Children’s Play Scale (CPS)
Source: BMC Public Health. 2021 May 7;21:878. doi: 10.1186/s12889-021-10812-x (PMC8103430; doi:10.1186/s12889-021-10812-x)
Supplement: Supplementary file 3 — Additional file 3. Numeric mappings. [file 12889_2021_10812_MOESM3_ESM.docx]

Table S5.

*Response mappings for how frequently parents responded that their child played in each place by season (Spring/Summer, Autumn/Winter). Play over the year period was calculated by summing the scores for Spring/Summer and Autumn/Winter.*

| **Response** | **Numeric mapping** |
| --- | --- |
| Every day | 182.5 |
| 4-6 days | 130 |
| 2-3 times per week | 65 |
| Once a week | 26 |
| Once a month | 6 |
| Less than once a month | 3 |
| Never | 0 |

Table S6.

*Response mappings for how much time parents responded that their child played in each place by season (Spring/Summer, Autumn/Winter). Time spent playing in each location was calculated by summing the scores for Spring/Summer and Autumn/Winter.*

| **Response** | **Numeric mapping** |
| --- | --- |
| Not applicable | 0 |
| Less than half an hour | 0.25 |
| Around an hour | 1 |
| 2-3 hours | 2.5 |
| 4+ hours | 5 |
